# Immunogenetic Association Underlying Severe COVID-19

**DOI:** 10.3390/vaccines8040700

**Published:** 2020-11-20

**Authors:** Kendall McCoy, Autumn Peterson, Yun Tian, Yongming Sang

**Affiliations:** 1 Department of Biology, College of Life and Physical Sciences, Tennessee State University, 3500 John A. Merritt Boulevard, Nashville, TN 37209, USA; kmccoy5@Tnstate.edu (K.M.); apeter15@Tnstate.edu (A.P.); 2 Department of Agricultural and Environmental Sciences, College of Agriculture, Tennessee State University, 3500 John A. Merritt Boulevard, Nashville, TN 37209, USA; ytian@tnstate.edu

**Keywords:** COVID-19, interferon signaling, chemokine signaling, genome-wide association, epigenetic regulation

## Abstract

SARS-CoV2 has caused the current pandemic of new coronavirus disease 2019 (COVID-19) worldwide. Clinical outcomes of COVID-19 illness range broadly from asymptotic and mild to a life-threatening situation. This casts uncertainties for defining host determinants underlying the disease severity. Recent genetic analyses based on extensive clinical sample cohorts using genome-wide association studies (GWAS) and high throughput sequencing curation revealed genetic errors and gene loci associated with about 20% of life-threatening COVID-19 cases. Significantly, most of these critical genetic loci are enriched in two immune signaling pathways, i.e., interferon-mediated antiviral signaling and chemokine-mediated/inflammatory signaling. In line with these genetic profiling studies, the broad spectrum of COVID-19 illness could be explained by immuno-pathological regulation of these critical immunogenetic pathways through various epigenetic mechanisms, which further interconnect to other vital components such as those in the renin–angiotensin–aldosterone system (RAAS) because of its direct interaction with the virus causing COVID-19. Together, key genes unraveled by genetic profiling may provide targets for precisely early risk diagnosis and prophylactic design to relieve severe COVID-19. The confounding epigenetic mechanisms may be key to understanding the clinical broadness of COVID-19 illness.

## 1. The Broad Spectrum and Critical Illness in COVID-19 Progression

The coronavirus disease 2019 (COVID-19), which has been declared a worldwide pandemic by the WHO since March of 2020, is caused by the novel coronavirus severe acute respiratory syndrome coronavirus 2 (SARS-CoV-2) [1,2,3,4]. The virus evolves at a highly contagious rate in human beings, with a basic reproduction number (R0) ranging at 1.4–5.7. The clinical outcome of COVID-19 varies broadly among infected people, ranging from asymptotic infection and common cold-like sickness to a severe pneumonia leading to acute respiratory distress syndrome (ARS) and multi-organ complications that potentially have fatal prognosis [5,6,7,8]. Complications of severe COVID-19 include vasculitis, coagulopathy, thrombosis, septic shock, and even multi-organ failure [5,6,7,8]. The epidemiology of COVID-19 shows a diverse pattern across people who are different in age, sex, ethnicity, and particularly among those with pre-existing medical conditions [6,7,8,9,10,11]. For example, the US statistics showed that older patients (aged ≥65 years) accounted for 31% of all cases, 45% of hospitalizations, 53% admissions of intensive care unit (ICU), and 80% of deaths, with the highest incidence of severe outcomes in patients aged ≥85 years [1,4,8]. Similarly, increased risk of critical and life-threatening illnesses was reported to associate with males and particularly pre-existing comorbidities, including cardiovascular, renal, liver, diabetes, and other autoimmune diseases as well as obesity condition [4,5,6,7,8,9,10,11]. In contrast, evidence indicates that children (median age 4–7 years) have a lower susceptibility and risk for critical illness. However, under the circumstance of comorbidity and genetic risks, the disparity of the risk for severe COVID-19 becomes vague concerning the factors of age, sex, and ethnicity [4,5,6,7,8,9,10,11]. With a critical viral disease like COVID-19, illness comes from both the virus infection and interacting with immune responses, especially a consequential imbalance of harmful immunopathies over proper immune responses. Upon exposure to the same virus, whereas individuals show asymptotic or mild illness plausibly mounting effective immune reactions, severe COVID-19 patients, however, may reflect dysfunctional immune reactions that further leads to pathological exacerbation accompanying uncontrolled virus spreading and immune overwhelming [9,10,11,12,13,14,15,16,17]. As the virological branch focuses on diminishing viral spreading and virulence to cause disease, deciphering the genetic and especially epigenetic associations underlie severe COVID-19 will grasp the immunogenetic theme for severity prognosis in the host, thus providing manageable targets for early risk diagnosis and development of prophylactic and therapeutic remedies to face current pandemic [18,19,20].

## 2. Genetic Association: Interferon and Chemokine Response Representing the Centric Immune Determinants Underlying Severe COVID-19

**Figure 1:**
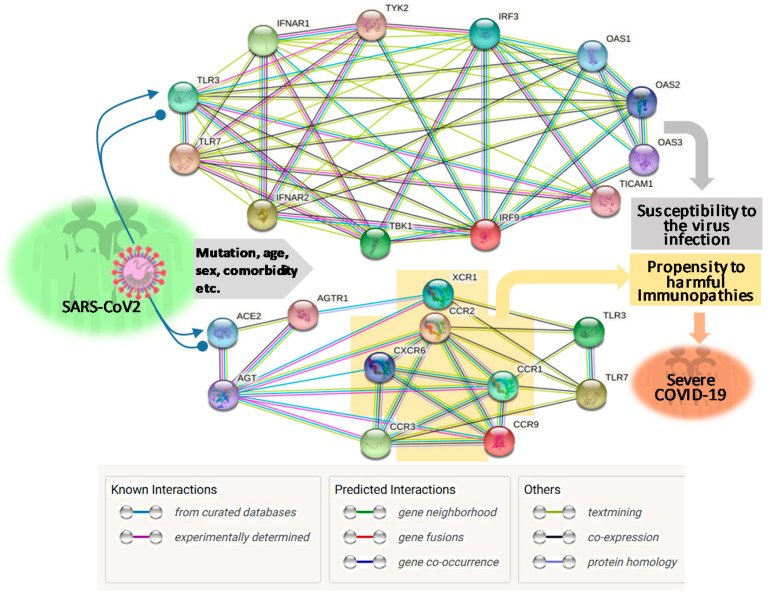
Interferon and chemokine signaling pathways are centrically enriched by key Immunogenetic determinants revealed by recent studies focusing on severe COVID-19 cohorts. Major genes associated with COVID-19 were pooled from several recent immunogenetic studies [22,23,24,31]. The protein-protein interaction networks were performed using a STRING program [20]. The centrically enriched chemokine receptor genes in Chr. 3p21.31, which are regulated by both RAAS and TLR signaling pathways critically in chemokine signaling of inflammatory response, is associated with severe COVID-19 by multiple GWAS studies and highlighted using a yellow cross. Abbreviations: ACE2, angiotensin-converting enzyme 2; AGT, angiotensin; AGTR1, AGTII receptor type 1; CCR, C-C chemokine receptor; CXCR, C-X-C chemokine receptor; IRF, IFN-regulatory factor; IFNAR, IFN-α/β receptor subunit; MAS1, OAS, 2′-5′-oligoadenylate synthase; TBK1, TANK-binding kinase 1; TICAM1, TLR, Toll-like receptor; TMPRSS2, transmembrane protease serine 2; TYK2, non-receptor tyrosine-protein kinase; XCR, Chemokine XC receptor.

About two months post the WHO declaring the COVID-19 pandemic, a global initiative of COVID-19 host genetics was commenced to elucidate the role of host genetic factors in SARS-CoV2 susceptibility and COVID-19 severity [21]. The first report about genome-wide association study (GWAS) of severe COVID-19 with ARS detected two genetic susceptibility loci at Chr3p21.31 and Chr9q34.2 using a meta-analysis of the two case/control panels including 835/1225 and 775/950 samples from Italy and Spain, respectively (Table 1) [22]. Significantly, the association within the locus Chr3p21.31 spans the genes SLC6A20, LZTFL1, CCR9, FYCO1, CXCR6, XCR1, CCR1, and CCR3 (gene symbols are standard ones from NCBI, see Figure 1 legend for definitions of abbreviations), which include several chemokine receptors (CCRs, CXCR6, and XCR1) mediating chemokine signaling pathways for leukocyte chemotaxis, inflammatory regulation and relevant immunopathies causing lung injury. Notably, Chr3p21.31 locus has been reproducibly associated with severe COVID-19 by at least three GWAS studies, indicating it constitutes a common genetic mechanism underlying severe COVID-19 [22,23,24]. Interestingly, an independent study also identified the ~50 kb region of locus Chr3p21.31 representing an allelic risk that was inherited from Neanderthals and is carried by ~50% of people in South Asia and ~16% of people in Europe today, who were predicted to be prone to the progression of severe COVID-19 [24]. In addition, the association of Chr3p21.31 locus was also reflected by the critical illness in the younger patients (<65 years) with less comorbidity, indicating a de facto genetic correlation [22,23,24]. Several clinical observations correlated blood types with the severity of COVID-19, i.e., O blood type seems more protective compared to a higher risk of non-O, especially A blood type [25,26,27]. One GWAS assay using two case-control European panels associated severe-COVID-19 with locus Chr9q34.2, which concurs the ABO blood group locus [22]. However, this association was not significantly demonstrated in two other GWAS assays published [23,24], indicating that the association of blood type locus with severe COVID-19 is not as universal as the chemokine receptor locus at Chr3p21.31 (Table 1). Therefore, more studies are needed to extensively verify the association of blood types with the progression of COVID-19 severity. In addition, no mechanistic research about the association of blood types with COVID-19 severity has been reported. In general, antigens determining blood types can serve as direct receptors or co-factors for some pathogenic infections; indirectly, many blood group antigens facilitate cell adhesion, substance intake and signaling transduction [22,25,26,27]. Given the reported inconsistency on association of blood types with COVID-19 severity, we interpret an indirect role (such as regulation through the RAAS system, see next section) of blood types on COVID-19 susceptibility and disease progression [22,25,26,27].

Pairo-Castineira et al. released their GWAS analysis using a bigger case/control cohort (2244/10220) from UK hospitals, which represent >95% of all ICU beds in the UK (Table 1) [23]. In addition to the detection of a strong association signal at the Chr3p21.31 locus, the study identified and replicated four novel genome-wide significant associations. These include: (1) at Chr6p22.1–33 region spanning major histocompatibility complex, class I-G, HLA-G, and Coiled-Coil Alpha-Helical Rod Protein 1, CCHCR1 genes; (2) at Chr19p13.3 locus within the gene encoding dipeptidyl peptidase 9 (DPP9); (3) at Chr12q24.13 locus spanning a gene cluster encoding antiviral restriction enzyme activators (OAS1, OAS2, OAS3); and (4) at Chr21q22.1 spanning the interferon receptor gene IFNAR2) [23]. Elegantly, the study also supplemented GWAS illumination with evidence using Mendelian randomization (MR) and transcriptome-wide association (TWAS) assays to define a causal link from the low expression of IFNAR2, and high expression of TYK2, to life-threatening COVID-19. TWAS in lung tissue determined the association of severe COVID-19 with increased expression of the monocyte/macrophage chemotactic receptor CCR2 [23]. Collectively, this study robustly determined genetic signals relating to key host antiviral defense mechanisms, especially that mediated by interferon (IFN)-signaling and chemokine receptors in orchestrating chemotactic and inflammatory responses as clinically demonstrated commonly in severe Covid-19 cases (Figure 1) [12,13,14,28,29,30].

Using an approach combining both next-generation sequencing (NGS) and experimental validation, Zhang et al. elucidated an enrichment of genetic risk variants at thirteen human loci governing the Toll-like receptor (TLR)-3- and IFN-regulatory factor (IRF)-7-dependent type I IFN immunity in 659 patients with life-threatening COVID-19 [31]. In contrast, few of these genetic risk variants were detected in the 534 control subjects with asymptomatic or benign infection. These 13 genetic risk loci displayed functional deficiency of these immune genes. They accounted for 3.5% of severe COVID-19 patients aged 17 through 77 years, and progressed to a life-threatening pneumonia without prior severe infection, indicating a determining role of dysfunctional IFN-mediated antiviral immunity underlying the progression of severe COVID-19 (Table 1 and Figure 1) [31].

## 3. Epigenetic Association: Undermined Interferon and RAAS Responses Leading to Antiviral Dysfunction, Hyperinflammation, and Autoimmunity

As discussed above, the inborn genetic errors in IFN signaling were associated with 3.5% life-threatening COVID-19 [31]. This, as the evidence representing natural loss-of-function genetic variants, verifies a determinant role of IFN signaling in the disease susceptibility and severity. On the other hand, these variants only represent a small portion of extremal examples that carry genetic deficiency incapable of mounting effective antiviral immunity. Logically, a large portion of severe COVID-19 cases may have resulted from deficiency or dysfunction of the biological mechanisms beyond the genetic DNA codes, i.e., at the epigenetic level that developmentally obtained from intricating gene expression networks upon various environmental situations experienced by different individuals [18,19,20].

One prominent feature of immunity is to distinguish self versus non-self and resist the invasion of non-self [32,33,34]. So, primary humoral antibodies are selectively against pathogenic antigens not reacting to self-antigens [32,33,34]; however, autoreactive antibodies (auto-Ab) that mistakenly target and react with self-antigens have been reported in patients with chronic infections and especially as a biomarker for various autoimmune diseases, such as rheumatoid arthritis, systemic lupus erythematosus (SLE), Sjogren’s syndrome, and multiple sclerosis [28,35]. Not only is the pre-existing autoimmune condition associated with a higher risk of severe COVID-19 incidences as repeatedly reported [28,35], but several studies also suggested that SARS-CoV2 infection and COVID-19 progression could potentially trigger an imbalance prone to the autoimmune and autoinflammatory response. The prevalence of auto-Abs has been reported in 20–50% of different COVID-19 patient cohorts [28,35]. Symptoms of severe COVID-19 patients resemble someway with the autoimmune diseases, including immune thrombocytopenic purpura (ITP), Guillian-Barrė syndrome (GBS), and Kawasaki like disease (KD) in terms of thrombosis, coagulopathy, chilblain, and vasculitis [28,35,36,37,38]. Significantly, auto-Abs identified in severe COVID-19 were evidently reactive to neutralize almost all type I IFN subtypes, thus preventing the major antiviral IFN action immunologically beyond the genetic scenario [39]. Bastard et al. identified the auto-Abs reactive to type I IFNs as an immuno-deficient cause for about 15% of 987 severe COVID-19 patients who had life-threatening pneumonia. These auto-Abs were primarily capable of neutralizing type I IFNs, mostly IFN-α and IFN-ω subtypes, which represent major circulatory IFN subtypes inducing systemic antiviral response and otherwise cause IFN-mediated immunopathies in a persistent infection or autoimmune condition [40,41,42,43]. By contrast, these IFN-targeting auto-Abs were not present in 663 patients with asymptomatic or mild COVID-19, and were only found in a few (4/1227) of healthy individuals as the control [39]. Reckoning the higher risk of severe COVID-19 in male sex, reactive auto-Abs were identified primarily in males (94%), indicating that IFN-targeting autoimmunity may contribute to higher incidence of severe COVID-19 in males [39]. The auto-Abs’ neutralizing activity diminished effective IFN peptides in the serum and functionally blocked IFN-mediated antiviral action. In addition, the vast presence of IFN-reactive auto-Abs indicates the persistence of type I IFNs, which generally correlates to an immunopathological response rather than a protective role during the late phase of the viral infection, such as in most cases of severe COVID-19 [40,41,42,43]. Notably, although the emerging of auto-Abs involves somatic hyper-mutation of the antibody genes in B cells, the sequential process for self-antigen selection and passing the checkpoint for antibody production should be mostly regulated at the epigenetic even post-translation levels, which is facilitated by an inflammatory or autoimmune condition commonly observed in severe COVID-19 progression [28,35,36].

**Figure 2:**
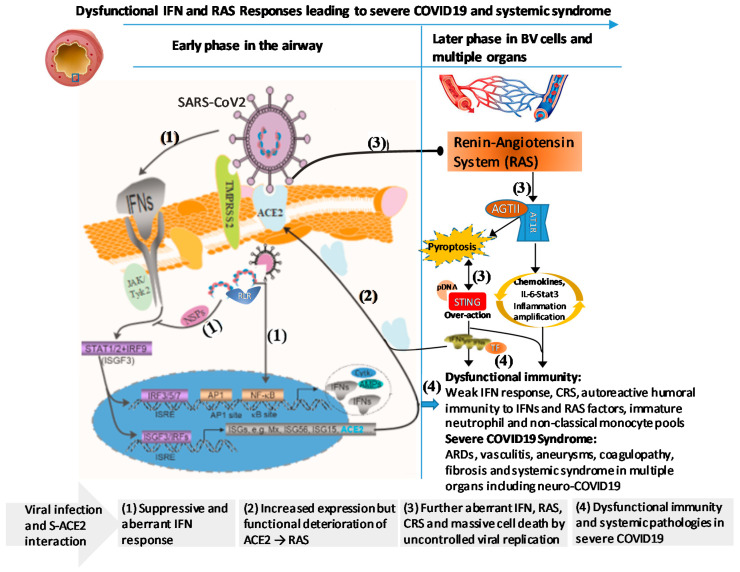
Dysfunctional interferon (IFN) and renin-angiotensin system (RAS) responses leading to severe COVID19. SARS-CoV2, the virus of COVID19, evolves in optimizing its infectivity and contagiousness to induce a weak antiviral innate immune IFN response during its acute phase of infection especially in the susceptible patients due to seniority, gender and existing medical conditions. SARS-CoV2 adopts angiotensin-converting enzyme 2 (ACE2), a critical and multirole component of the body RAS, as a major viral receptor expressed in multiple cell types. The progressive infection of the virus from pneumocytes to BV cells exaggerates RAS imbalance provoking proinflmmation, CRS and massive cell death (pyroptosis) catastrophized with viral spreading, which further provokes coagulopathy/fibrosis (as in Kawasaki syndrome) and autoimmune reactions against IFNs and RAS components. All these collectively lead to a complicated syndrome including systemic virus infection in multiple organs and functional deterioration of RAS plus compounding immunopathies and neurological manifestation. Abbreviations: AGTII, angiotensin II; AP1, activator protein 1 transcription factor; ARD, acute respiratory disease; AT1R, AGTII receptor type 1; BV, blood vessel; CRS, cytokine release syndrome; ISRE, IFN signaling responsive element; ISGF3, IFN-stimulated gene factor 3; NSP, non-structural proteins; RLR, retinoic acid-inducible gene-I-like receptors; pDNA, pathogenic DNA; STING, stimulator of interferon genes; TF, tissue factor.

The SARS-CoV2 virus evolves to adapt its deadliness and contagiousness for efficient spreading in humans [6,7]. The viral infection causes substantial differences in susceptibility and disease severity in people of different ages, genders, and preexisting comorbidities [8,9,10,11]. For the severe COVID-19 cases, except the ~19% with life-threatening pneumonia that is caused by IFN-deficiency functionally through genetic and autoimmune errors as described above [31,39], the severity progression in the other majority of severe COVID-19 patients may be underlain by epigenetic regulation of host factors connected to the IFN and chemokine signaling pathways as genetically elucidated [22,23,24]. Epigenetic regulation, which molecularly acts on gene expression through DNA methylation, histone modification, and regulatory RNA operation, provides a dynamic scenario and toolkit to understand the outcome broadness of COVID-19 [18,19,44,45,46]. Few studies have directly examined epigenetic regulation in SARS-CoV2 infection and the progression of severe COVID-19 in the host [20,45,46,47]. Collective evidence indicates that epigenetic mechanisms play an important role in the severity progression of COVID-19 [45,46,47] and regulate the overall process from the virus initial interaction with its primary cell receptor, angiotensin-converting enzyme 2 (ACE2), to the complication into severe illness [20,44,45,46,47]. Indeed, dysregulation of IFN, chemokine, and other immune pathways have been correlated with aging, gender difference, and exceedingly various comorbid conditions, which have been recently observed for association with a higher risk of severe COVID-19 [8,9,10,11]. For examples, compared with juveniles, the secretion of both type I and type III IFNs by dendritic cells (DCs) in the blood or lung is severely impaired in aged individuals; by contrast, blood DCs from aged individuals produce higher basal levels of proinflammatory cytokines/chemokines including interleukin-6 (IL-6), C-X-C motif chemokine-8 (CXCL-8), CXCL-10, and tumor necrosis factor (TNF-α) [48,49]. This aging-associated aberrancy in DC response, together with the other observation of neutrophilia [50], invokes lung inflammation, impair antiviral resistance and exaggerates major clinical signs as observed in severe COVID-19 [1,2,3,4,5,6,7]. For most preexisting comorbidities such as hypertension, cardiovascular diseases, or diabetes mellitus that increase the risk of severe COVID-19 [51], various studies have shown the progressive association of IFN insensitivity and chemokine/cytokine-mediated chronic inflammation and have been reviewed elsewhere [41,42,43,44]. In addition to the autoimmune reaction incited by SARS-CoV2 infection, immunopathies from IFN-persistence, inflammation, and specifically auto-Abs represent typical pathological mechanisms underlying most preexisting autoimmune diseases such as SLE, diabetes, and sclerosis [41,42,43,44]. Auto-Abs, which bookmark different autoimmune diseases, may target self-antigens, including critical cytokines like IFNs [39,52,53,54]. The prevalence of auto-Abs against innate immune IFNs in life-threatening COVID-19 patients indicates an autoimmune ambient accompanied by an overwhelmed IFN response dysregulated by pathogenic DNA from massive cell death caused by the robust virus infection, which is mediated through a cyclic GMP–AMP synthase (cGAS) and signaling effector stimulator of interferon gene (STING) pathway (Figure 2) [55,56,57,58]. For the gender difference, Webb et al. recently reported that plasmacytoid dendritic cells (pDC) from healthy females, especially after puberty, produced more type I IFNs via TLR7-mediated signaling than males [59,60]. This finding indicates that the inferiority of males in the early antiviral IFN induction, an adequate period demonstrated by most IFN-based clinical trials on combating the viral infections [61]. The study also identified that this sex-associated difference in IFN production is related to ChrX number and serum testosterone concentration [59]. Because no loci in ChrX has been genetically associated with severe COVID-19 [21,22,23,24], we interpret that SARS-CoV2 binding and inducing ACE2 degradation may indirectly cause gender difference involving both renal endocrine and immune responses through the renin-angiotensin-aldosterone system (RAAS) axis [62,63,64]. Many studies have indicated the critical role of the virus-ACE2 interaction in disease susceptibility and disruption of RAAS in disease severity progression [65,66,67,68,69,70]. The key points emphasize that: (1) evolutionary affinity of SARS-CoV2 to ACE2 determines the virus-cell permissiveness and host species tropisms [20,65]; (2) the binding of the viral spike protein (S) to ACE2 inhibits and disrupt angiotensin (Ang) conversion and relevant Ang humoral homeostasis; and (3) the RAAS is then diverted to physio-pathological induction of vasoconstriction, hypertension, fibrosis, oxidative stress, and proinflammation [62,63,64,65,66,67,68,69,70,71]. Cross-pathway component analysis indicates that the key chemokine-receptor loci on Chr3 are intricately connected to both TLR3/7-mediated immune signaling and RAAS signaling (Figure 1). This indicates that whereas dysregulated IFN-signaling may determine the disease susceptibility [12,13,14,15], hyperinflammation signified by an overreaction of chemokine signaling is intersected by both immune and pathophysiological regulation through RAAS [55,56,57,58,68,70] (Figure 1 and Figure 2). 

**Table 1:** Genetic inborn or epigenetic obtaining errors associated with severe COVID-19 *.

Chr. Location (Key Genes Covered, or Epigenetic Effect)	Association (Appr./OR: Freq.)	Major Immune Pathway Involved	References & Notes
3p21.31 (SLC6A20, LZTFL1, FYCO1, CXCR6, XCR1, and CCR9; Neanderthal-originated allelic region)	GWAS 95% CI/ (1.95–2.79: 1610 vs. 2205) [22] (2.14: 2244 vs. ~5X 2244) [23]	ACE2 mediated amino acid transport (SLC6A20); Chemokine and Inflammation signaling, chemotaxis, immunopathies for lung injury (others)	Associated at [22,23,24]
6p22.1–33 (HLA-G, CCHCR1, NOTCH4)	GWAS 95% CI/ (1.30–1.85: 2244 vs. ~5X 2244)	Antigen processing and presentation (HLA); P-body component for RNA metabolism, associated with psoriasis (CCHCR1); lymphocyte development (NOTCH4)	Associated by [23]
9q34.2 (ABO blood type locus)	GWAS 95% CI/ (1.37–1.45: 1610 patient vs. 2205 control)	Blood type-dependent pathological reaction, such as coagulation and thrombolysis	Associated by [22]
12q24.13 (OAS1, OAS2, OAS3)	GWAS 95% CI/ (1.29: 2244 vs. ~5X 2244) [23]	IFN-mediated antiviral signaling	Associated by [23]
19p13.3 (DPP9, TYK2)	GWAS 95% CI/ (1.36–1.59: 2244 vs. ~5X 2244) [23]	Innate antiviral defense (TYK2), and antigen presentation, CXCL10 signaling, and associated to obesity, diabetes, and cancer (DPP9)	Associated by [23]
21q22.1 (IFNAR2)	GWAS 95% CI/ (1.28: 2244 vs. ~5X 2244) [23]	IFN-mediated immune signaling	Associated by [23]
Several Chr. (TLR3, UNC93B1, TICAM1, TRAF3, TBK1, IRF3/7/9, IFNAR1/2, STAT1/2)	NGS and variant calling, wet-bench validation (3.5% of 659 severe COVID-19 vs few in 534 control)	IFN mediated immune signaling	Detected by [31]
Epigenetic obtaining (Autoantibody against IFNs, 94% in male)	Wet-bench detection (13.7% of 987 severe COVID-19 vs. 0.33% in 1227 control)	IFN mediated immune signaling	Detected by [39]
Epigenetic obtaining (Higher incidence of severe COVID-19 in aged, male, and comorbid patients)	Inclusive studies and evidence (Higher incidence of severe COVID-19 in aged, male, and comorbid patients)	Dysregulated IFN and chemokine responses, chronic/systemic inflammation, impaired other immune responses	Exemplified by [18,19,20,39,44,45]

* Defined as accompanying respiratory failure in hospitalized patients. Abbreviation: Appr., approaches to associate the gene loci with severe COVID-19 in the references; CI, confidence intervals; Chr., human chromosome from genome build hg38; GWAS, genome wide association study; IGV, integrative genomics viewer; NGS, next-generation sequencing; OR: Freq., odds ratio: frequency in severe COVID-19 patient vs in the control groups. The gene symbols are standard ones from NCBI, see Figure 1 legend for definitions.

Correspondingly, hypercytokinemia has been observed in severe COVID-19. Inflammatory mediators, including calprotectin (S100A8/9), CRP, IL-1, IL-10, and TNF-α are increased 2–100 fold, whereas IL-6 can be elevated more than 1000 fold above normal in reported cases [13,71,72,73,74,75]. Several cohort studies reported that markedly elevated serum IL-6 levels in the 100–10,000 pg/mL range in severe COVID-19 patients [13,73,74,75]. Of 15 clinical parameters diagnosed at hospital admission, elevated CRP (at a cutoff of 87.5 mg/L) and IL-6 levels (at a cutoff of 86 pg/mL) were significantly correlated to death prediction [74]. Clinically reflected in life-threatening respiratory failure in COVID-19, elevated serum IL-6 is also associated with lymphopenia, functional T-cell deficiency, and vasculitis [71]. Likewise, in severe COVID-19, various biomarkers of immune dysregulation exacerbate to a common terminal hyperinflammation indicated by robust incidence of IL-6 and lymphopenia accompanying respiratory failure, which may directly or indirectly intersect to the dysfunctional IFN- and chemokine-signaling pathways that are significantly enriched by the genetic associations [13,21,22,23,24,71,72,73,74,75]. In addition, these findings suggest severe COVID-19 may be viewed as an inflammatory vasculitis, which is induced post the viral infection of pneumocytes, endothelial and epithelial cells, and the viral suppression of the RAAS system [71,72,73,74,75]. These may rear an epigenetic ambient, leading to inflammatory and immunopathic consequence associated with severe COVID-19 progression locally in the lung or systemically in multiple organs. As a piece of supporting evidence, numerous drug-repurposing studies of cytokine blockade (such as using anakinra and tocilizumab) and JAK inhibition (such as using baricitinib) have shown promise [71,72,73,74,75]. A relevant proposal is that targeting management of epigenetic regulation may provide valuable approaches to relieve inflammatory and immunopathic overdrive in severe COVID-19 [76]. In summary, GWAS and NGS profiling unravel the significant association of severe COVID-19 with multiple genetic errors at the gene loci enriching in IFN and chemokine signaling pathways [22,23,24,31]; however, the immuno-pathological determinants for the other major part of severe COVID-19 cases may be complicated by the interaction of the viral and especially host factors at the epigenetic levels [18,19,20,44,45,46,47,48,57]. The IFN- and chemokine-centric determinants verified through the systemic genetic approaches confer critical nodes to decipher host immune mechanisms underlying the severity progression in COVID-19, thus facilitating host-originated designs for early risk diagnosis and repurposing prophylactic/drugs for mitigating severe COVID-19 [22,23,24,31,39,67]. 

## 4. Conclusions Remarks: Precise Early-Risk Diagnosis and Drug Repurposing for Severe COVID-19 Based on Immunogenetic Association

Clinical outcomes of SARS-CoV2 infection are extensively broad in complication further with symptoms mimicking various inflammatory and autoimmune syndromes in severe COVID-19. Remarkably, most of these symptomatic signs reflect immuno-pathological regulation through the epigenetic mechanisms, which facilitate us to explain the broadness and the dynamics of the clinical outcomes but shadow the focus of key immune determinants triggering the severity of COVID-19 [18,19,20,44,45,46,47,48,57]. GWAS and NGS profiling, which pursue genetic commons shared by the big cohorts of clinical samples, thus enable to probe determining factors owing to genetic variance behind the environmental variables (mostly through epigenetic regulation) which complicates the illness outcome at various extents [21,22,23,24,44,45,46,47,48,57]. Thus, these genetic risk factors comprise a panel of biomarkers for precise early diagnosis and precaution of severity in relevant people before the infection and disease progression. The elucidation of these genetic risk loci that enrich in IFN- and chemokine-signaling also direct the critical targets for prophylactic designs through either new drug invention or repurposing the existing drugs [77,78,79,80,81]. Notably, type I IFN-signaling represents a critical antiviral innate immunity, which is favorable primarily at the early phase of viral infection prior to progression into a severe situation [61,77]. Accordingly, most prophylactic IFN applications, but not recent therapeutic IFN-based trials of severe COVID-19, are likely more promising [61,77]. For the treatment of hospitalized severe cases of COVID-19, interventions targeting the RAAS imbalance and chemokine/inflammation exaggeration seem more effective (Figure 2). For instance, a recent clinical study reported that intravenous delivery of a recombinant human soluble ACE2 (hrsACE2) for seven days relieved the illness of a severe COVID-19 patient, showing the suppression of inflammatory biomarkers, reduction of viral load, and increase of Ang II and virus-neutralizing antibody production [82]. Although genetic loci in the RAAS pathway have not been associated with severe COVID-19 [21,22,23,24,31], this study suggests that the components of RAAS could be physio-pathological determinants underlying severe COVID-19 because ACE2 is directly adopted by SARS-CoV2 for infection and RAAS exerts functionally crosstalk to immune response and regulation such as intersecting to chemokine/inflammatory pathways [65,66,67,68,69,82].
